# Ulcerative colitis-associated diffuse enteritis without prior colectomy: A case report

**DOI:** 10.1097/MD.0000000000042924

**Published:** 2025-06-20

**Authors:** Qiuming He, Wanhui Wei, Jie Li, Zhitao Chen, Heng Zhang

**Affiliations:** a Department of Gastroenterology, The Central Hospital of Wuhan, Tongji Medical College, Huazhong University of Science and Technology, Wuhan, China; b Key Laboratory for Molecular Diagnosis of Hubei Province, The Central Hospital of Wuhan, Tongji Medical College, Huazhong University of Science and Technology, Wuhan, China.

**Keywords:** biologic therapy, extraintestinal manifestations, infliximab, small bowel inflammation, ulcerative colitis

## Abstract

**Rationale:**

Ulcerative colitis (UC) is a chronic inflammatory bowel disease that primarily affects the colon. Although UC is classically limited to the colonic mucosa, rare cases of small bowel involvement have been reported. UC-associated small bowel inflammation is an uncommon complication, most frequently described following colectomy with ileal pouch formation. However, emerging evidence suggests that it can also occur in patients without prior surgery.

**Patient concerns:**

A 27-year-old female with a 6-year history of UC, well-controlled with mesalazine, developed intermittent periumbilical and left upper quadrant abdominal pain 5 years after disease onset.

**Diagnoses:**

Small bowel endoscopy revealed the presence of multiple ulcers in the horizontal duodenum and proximal jejunum. Histopathological examination showed loss of villous epithelium, widespread hemorrhage in the lamina propria, flattened crypts, reduced crypt density, and neutrophilic infiltration with associated bleeding.

**Interventions:**

After the diagnosis of UC-associated small bowel inflammation was confirmed through imaging, endoscopy, and histopathological examination, the patient did not respond to steroid therapy. Treatment with infliximab resulted in significant clinical improvement.

**Outcomes:**

The patient’s abdominal pain and inflammatory markers significantly improved after infliximab therapy. Follow-up endoscopy confirmed mucosal healing. She remained in steroid-free clinical remission during follow-up.

**Lessons:**

UC-associated small bowel inflammation is typically observed following colectomy; however, this case underscores that it can also occur in patients without prior colectomy. The condition often presents with unexplained abdominal pain, nausea, and vomiting, symptoms that may not be attributable solely to UC. For patients with extraintestinal symptoms who do not respond to steroid treatment, biologic therapies, such as infliximab, may provide an effective therapeutic option.

## 1. Introduction

Ulcerative colitis (UC) is a chronic idiopathic inflammatory bowel disease characterized by continuous mucosal inflammation of the colon. Although the disease primarily affects the colon, up to 24% of UC patients develop extraintestinal manifestations (EIMs), which can significantly contribute to disease burden and complicate management strategies.^[1,2]^

EIMs associated with UC encompass a wide spectrum of organ systems. The musculoskeletal system is most commonly involved, manifesting as peripheral arthritis or axial arthropathies, such as ankylosing spondylitis.^[3]^ Dermatologic EIMs include erythema nodosum, leukocytoclastic vasculitis, and pyoderma gangrenosum.^[4,5]^ Ocular manifestations, though less frequent, can range from episcleritis and scleritis to uveitis, and may precede intestinal symptoms.^[6]^ In addition, hepatobiliary involvement, particularly primary sclerosing cholangitis, is a well-recognized comorbidity in UC and is associated with a distinct disease course.^[7]^

UC-associated small bowel inflammation is a rare and often under-recognized complication, typically observed in patients following colectomy.^[8–10]^ However, recent reports indicate that patients without prior colectomy may also develop small bowel involvement. This condition is frequently challenging to diagnose due to nonspecific symptoms, including abdominal pain, nausea, and vomiting, which may overlap with those of other gastrointestinal disorders.^[11]^

In recent years, biologic therapies, particularly anti-TNF-α monoclonal antibodies such as infliximab,^[12]^ have demonstrated efficacy in the treatment of UC-associated small bowel inflammation. These biologic agents can effectively control inflammation in both the colon and small bowel, particularly in patients who do not respond to conventional therapies, including steroids. This case report presents a 27-year-old female with UC-associated small bowel inflammation, who was successfully treated with infliximab following failure of steroid therapy.

## 2. Case presentation

### 2.1. Chief complaints

A 27-year-old female with a 6-year history of UC, well-controlled with regular mesalazine therapy, presented with a 1-year history of intermittent periumbilical and left upper quadrant abdominal pain. The pain gradually intensified, accompanied by nausea and weight loss.

### 2.2. History of present illness

The patient was diagnosed with UC at the age of 21 and had been receiving mesalazine therapy, which effectively controlled her symptoms. However, 5 years after diagnosis, she began experiencing worsening abdominal pain. The pain was localized to the periumbilical and left upper quadrant regions, accompanied by nausea and weight loss. There was no history of diarrhea or rectal bleeding, and the UC symptoms remained well-controlled with mesalazine therapy. Despite this, the abdominal pain and associated symptoms did not improve. Physical examination revealed mild weight loss and tenderness in the left upper quadrant and periumbilical regions. No palpable masses or signs of peritoneal irritation were observed.

### 2.3. Past medical history

The patient had a history of UC but no other significant medical conditions. There was no history of gastrointestinal surgeries or known drug allergies.

### 2.4. Laboratory and imaging studies

**Figure 1.**
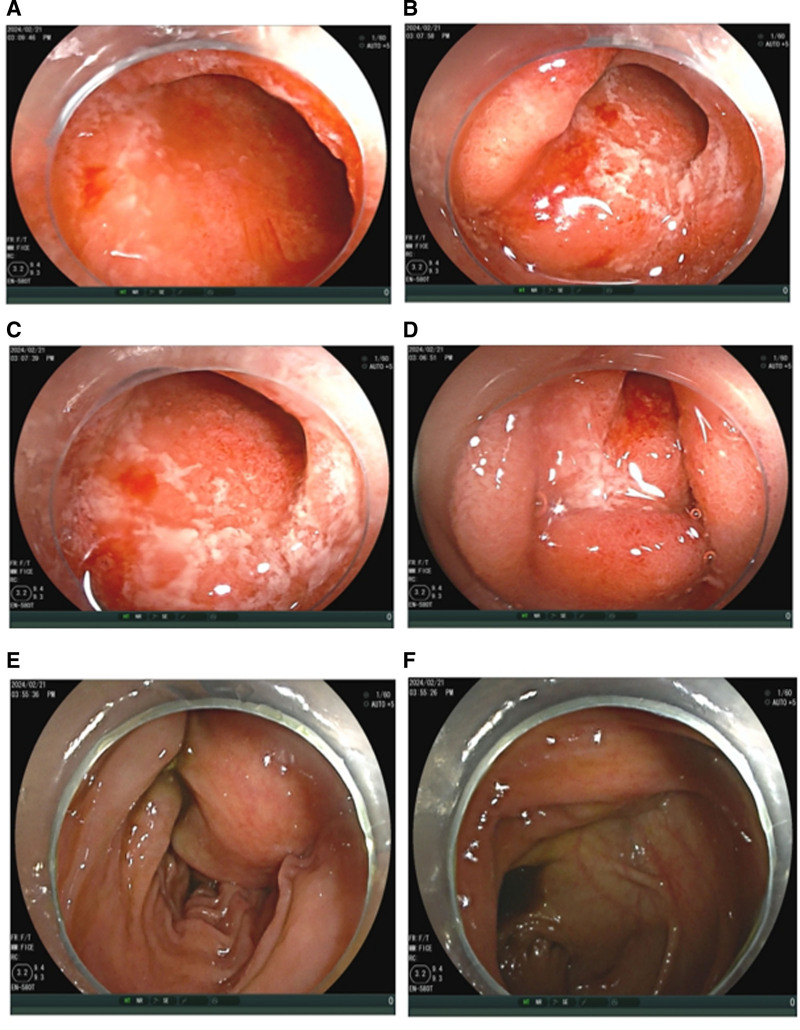
(A, B) Endoscopic image of the horizontal duodenum under small bowel enteroscopy. (C, D) Endoscopic image of the jejunum under small bowel enteroscopy. (E, F) Endoscopic image of the ileum under small bowel enteroscopy.

Small bowel endoscopy: Small bowel endoscopy revealed multiple shallow ulcers in the horizontal duodenum and proximal jejunum, accompanied by granular mucosal changes, edema, and congestion (Fig. 1).

**Figure 2.**
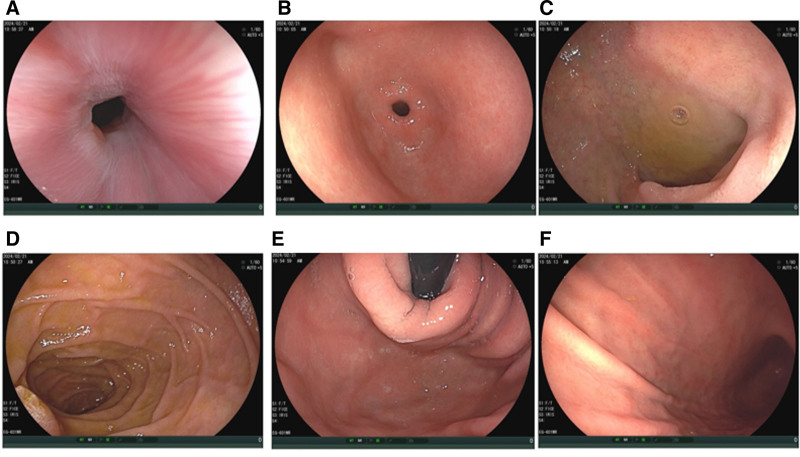
(A–F) Endoscopic image of the cardia, fundus, body, angulus, antrum, and pylorus, respectively.

Gastroscopy: No significant abnormalities were observed during the procedure (Fig. 2).

**Figure 3.**
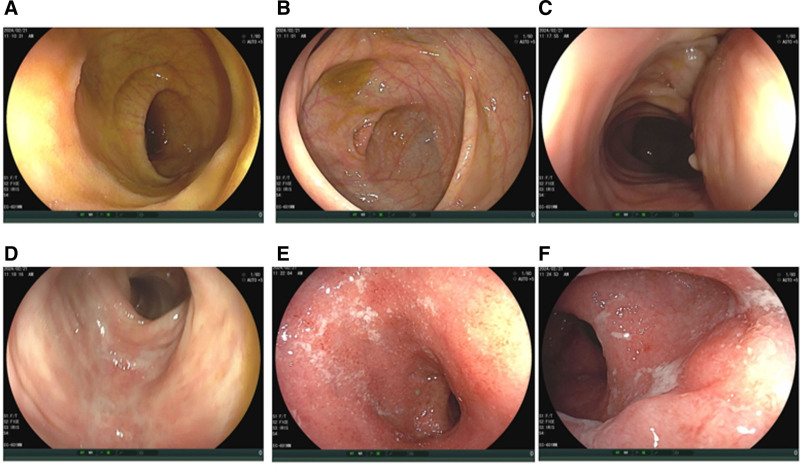
(A–F) Representative pictures from colonoscopy.

Colonoscopy: The appendiceal orifice, cecum, and ascending colon showed mucosal hyperemia and edema, with scattered erosions. The descending colon, sigmoid colon, and rectum exhibited normal lumen morphology, but mucosal hyperemia and edema were present, with blurred vascular patterns, scattered erosions, and shallow ulcerations. There was also significant fibrinous exudate (Fig. 3).

**Figure 4.**
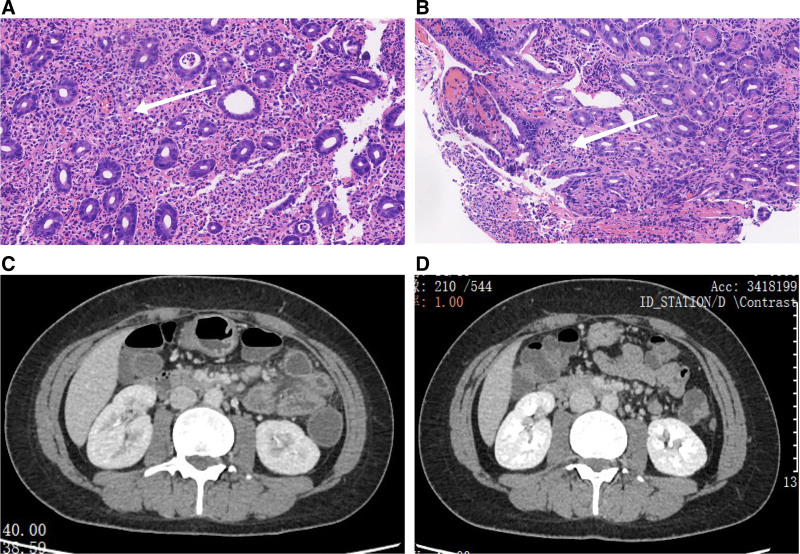
(A) Histopathological examination of jejunal ulcer. Biopsy revealed loss of villous epithelium, widespread hemorrhage in the lamina propria, and neutrophilic infiltration with associated bleeding (highlighted by arrow). (B) Histopathological examination of jejunal ulcer. The crypts were flattened with reduced density, and there was evidence of crypt inflammation and crypt abscesses (highlighted by arrow). (C) Intestinal CT images before treatment before with infliximab. (D) Intestinal CT images before treatment after with infliximab.

Histopathological examination: Biopsy revealed loss of villous epithelium, widespread hemorrhage in the lamina propria, and neutrophilic infiltration with associated bleeding. The crypts were flattened with reduced density, and there was evidence of crypt inflammation and crypt abscesses (Fig. 4A, B).

Imaging: The walls of the left mid-abdominal small intestine, splenic flexure, sigmoid colon, and rectum show mild thickening. The number of lymph nodes in the peri-intestinal and mesenteric regions increased, with some appearing to be slightly enlarged. Exudation was observed in the surrounding spaces and inflammatory changes were suspected (Fig. 4C, D).

Based on imaging, endoscopic findings, and histopathological results, the patient was diagnosed with UC-associated small bowel inflammation.

### 2.5. Treatment and management

The patient initially received intravenous corticosteroid therapy; however, her symptoms did not improve substantially. Given the lack of response to steroids, treatment was switched to infliximab (5 mg/kg intravenously). After 6 weeks of treatment, the patient reported significant relief from abdominal pain and other associated symptoms, including nausea and weight loss. Follow-up small bowel imaging revealed a marked reduction in inflammation (Fig. 1D), and her C-reactive protein and fecal calprotectin levels returned to normal. Given the positive response to infliximab, the patient continued biologic therapy and mesalazine was gradually tapered. She remained in clinical remission, with no recurrence of abdominal pain or other UC-related symptoms.

## 3. Discussion

UC is classically confined to the colonic mucosa. However, a growing body of evidence indicates that UC may, in rare instances, involve more proximal segments of the gastrointestinal tract, including the small intestine and even the upper gastrointestinal tract.^[1]^ UC-associated small bowel inflammation is a rare and frequently underdiagnosed complication of UC.^[13–15]^

For example, Muran Li et al^[16]^ described duodenal inflammation post-colectomy, with lesions ranging from superficial ulcers to diffuse erosive duodenitis. One of the cases resulted in duodenal necrosis and septic shock, underscoring the potential severity of this manifestation. Similarly, Terashima and colleagues reported 2 patients with UC-associated duodenitis in the absence of Crohn disease, both responding well to corticosteroids, suggesting that such lesions may be steroid-responsive manifestations of UC.^[17]^ This contrasts with our case, where the patient was steroid-refractory but responded to infliximab, highlighting variability in treatment response and the potential role of biologics in managing such atypical disease extensions. Moreover, Sietske Corporaal et al^[9]^ identified small intestinal inflammation shortly after colectomy in UC patients. Histologically, these lesions demonstrated epithelial apoptosis and lacked features typical of Crohn disease, such as granulomas. These findings support the hypothesis that UC-associated enteritis is a distinct entity rather than misclassified Crohn disease. Our patient similarly exhibited histological features consistent with UC, such as crypt distortion and neutrophilic infiltration, but no granulomas or transmural inflammation.

However it can also develop in patients without prior surgical intervention. Our patient presented with proximal small bowel involvement (duodenum and jejunum) without prior colectomy, a presentation that remains exceedingly rare. Notably, unlike the majority of published cases, our patient had no history of bowel surgery. This supports the notion that small bowel involvement can occur independently of surgical manipulation, possibly due to immune dysregulation extending beyond the colon in a subset of UC patients.

The clinical presentation of UC-associated small bowel inflammation may include abdominal pain, nausea, vomiting, and weight loss, symptoms that can mimic other gastrointestinal disorders. As a result, this condition can be challenging to diagnose without appropriate imaging and endoscopic evaluation.

In recent years, biologic therapies, particularly anti-TNF-α monoclonal antibodies such as infliximab, have demonstrated efficacy in the treatment of UC-associated small bowel inflammation. These agents act by inhibiting TNF-α, a cytokine involved in the inflammatory process, and have been shown to reduce inflammation in both the colon and small bowel.^[18]^ The use of biologic therapy is particularly beneficial for patients who do not respond to conventional treatments, such as corticosteroids.

This case underscores the importance of considering UC-associated small bowel inflammation in UC patients with unexplained abdominal pain or other gastrointestinal symptoms. Early diagnosis and treatment with biologic agents may improve patient outcomes and help prevent the progression of the disease.

## 4. Conclusion

UC-associated small bowel inflammation is a rare complication that can occur in UC patients, even without prior colectomy. The condition presents with nonspecific symptoms, including abdominal pain, nausea, and vomiting, which may be difficult to differentiate from those of other gastrointestinal disorders. Biologic therapies, particularly infliximab, provide an effective treatment option for patients who do not respond to conventional therapies. Clinicians should consider UC-associated small bowel inflammation in UC patients with unexplained abdominal symptoms and administer biologic therapy when conventional treatments fail.

## Author contributions

**Conceptualization:** Jie Li, Zhitao Chen.

**Data curation:** Wanhui Wei.

**Writing – original draft:** Qiuming He.

**Writing – review & editing:** Heng Zhang.
